# Modern contraceptive utilization and associated factors among younger and older married youth women in Ethiopia: Evidence from Ethiopia Mini Demographic and Health Survey 2019

**DOI:** 10.1371/journal.pone.0300151

**Published:** 2024-05-28

**Authors:** Kedir Abdu Yesuf

**Affiliations:** Department of Health informatics, Dessie Health Science College, Dessie, Ethiopia; Jimma University, ETHIOPIA

## Abstract

**Introduction:**

Utilization of modern contraceptives increases over time but it was still low and varies across ages among married youth woman. This study revealed the prevalence of modern contraceptives and its associated factors among younger and older married youth women.

**Methods:**

A cross-sectional study design was applied to the sample of EMDHS 2019. Multilevel logistic regressions were carried out using STATA version 16 to identify the individual and community-level factors of modern contraceptive utilization. Adjusted odds ratios with a 95% confidence interval and variables with a *p*-value < 0.05 were considered to be significant determinants of modern contraceptive utilization.

**Result:**

In the EMDHS 2019, a total of 3290 married women between ages 15 and 34 were included. Among these 1210 (36.7%) and 2080 (63%) women, they were age groups of 15–24 and 25–34 years, respectively. Modern contraceptive utilization among women aged 15–24 and 25–34 years was 54.23% and 52.6%, respectively. Injection is a commonly used modern contraceptive method. In this study, factors associated with modern contraceptive utilization among women aged 15–24 years include women who had primary education [AOR = 2.22; 95% CI: 1.02–4.83], who had three or more children in the household [AOR = 14.29; 95% CI: 1.61–126.25], Protestants [AOR = 0.29; 95% CI: 0.14–0.61], five to seven households [AOR = 0.34; 95% CI: 0.17–0.69], and region [AOR = 6.98; 95%:2.30–21.16]. On other hand, factors associated with modern contraceptive utilization among women aged 25–34 were women who had one or two under-five children in the household [AOR = 1.66; 95% CI: 1.03–2.68] and region [AOR = 3.54; 95%CI: 1.79–6.97].

**Conclusions:**

More than 50% of participants used modern contraceptives in both age groups and, the associated factor of modern contraceptive utilization varied among this age group. Health managers and policymakers need to consider age group, region, educational status, religion, and fertility level in planning of family planning program.

## Introduction

Family planning is the capability of individuals and couples to anticipate and attain their desired number of children and the spacing and timing of their birth, which is achieved through the use of contraceptive methods [1]. Family planning is a cost-effective way to reduce maternal mortality by reducing the number of high-risk births. Family planning also benefits by spacing births, easing pressures on the family finances, and giving time for income generating activities [2].

Modern contraceptive methods were the most common family planning method, accounting 92% of all contraceptive methods [3]. Modern contraceptive methods include male and female sterilization, male and female condoms, depot implants, pills, lactation amenorrhea method (LAM), Intrauterine devices (IUD), and emergency contraception [4].

Contraceptives are intended to slow the rate of population growth, particularly in developing countries [5]. Globally, the population grows rapidly, reaching 8 billion in 2023. Ethiopia ranks 10th in fastest-growing population in the world and is the second-most populous nation in Africa due to its low utilization of contraceptives [6, 7]. High fertility impedes opportunities for economic development, increases health risks for women and children, and erodes the quality of life by reducing access to education, nutrition. So contraceptives have a great contribution to the development of a country at all [8].

According to the United Nations, youth are defined as people between the ages of 15 and 24 years, but in the African Union and many other African nations, including Ethiopia, youth are defined as those aged between 15 and 34 years [9, 10]. These young people faced many maternal problems related to childbirth [11, 12]. As well, large numbers of young people in developing countries have more children than they desire, and their children face a high risk of low birth weight, preterm delivery, and severe neonatal conditions. Therefore, this can all be prevented by using contraceptive methods [11, 13].

Modern contraceptive utilization among younger and older youth women is crucial for avoiding unwanted pregnancy and unsafe abortion [14]. Contraceptives contribute to expanding education opportunities, empowering women, and reducing poverty. Overall, contraceptive use among youth is critical to improving the health, social, and economic outcomes for future generations [15]. unfortunately, youth in developing regions had lower levels of education, lived in rural areas, and resided in poorer households, which resulted in lower levels of contraceptive use [16].

Supporting youth access to and use of contraception can reduce maternal deaths by 40%. In addition, contraception also reduces infant mortality by 10% and childhood mortality by 21%. Therefore, modern contraceptives help youth plan their family size more effectively [1]. It is attained by implementing effective policies and programs towards family planning goals for youth [17, 18].

Globally, 25% of all young women use modern contraceptives. The use of modern contraceptives among youth has increased significantly over the last two decades [14]. However, contraceptive use among youth was lower than among older women in developing countries [19]. Due to discrimination, stigma, and a lack of information, many young people face barriers to accessing modern contraception [20–22]. Contraceptive use among young people aged 15 to 35 is associated with insufficient access to health information [23]. Contraceptive use among youth is also affected by the level of method failure and method discontinuation [24].

Married young women had a lower percentage of demand satisfied by a modern contraceptive compared with unmarried young women. Most young women want to have a child once they get married, and they want to demonstrate their fertility [25, 26]. Married young women who wish to delay their next pregnancy face several challenges in their decision-making. Previous studies showed that women are under pressure to have children once they get married due to traditional beliefs and male partners [25, 27, 28]. Young unmarried women use contraceptives to avoid pregnancy because of the social stigma associated with early pregnancy and because they have no income to support a child on their own. Some married women prefer to use contraceptives after giving birth to several children, but these results in economic challenges to raising the children. So married women are less empowered to decide on their own to adopt contraceptives [27, 29].

Modern contraceptive utilization is not even distributed; there is a different degree of inequality in contraceptive utilization that varies greatly among countries. Similarly, modern contraceptive use varied significantly by age across the country. Significant variations in the prevalence of modern contraceptive utilization and inequality within different age groups were discovered as a result of many factors related to it, such as the individual-level and community-level factors at the country level [30, 31].

Various individual-level factors influence the use of modern contraceptives, such as education status, income, wealth index, religion, and age at first birth [23, 29, 32, 33]. As well, the number of living children in a household, parity, family size, and births in the last three years affect modern contraceptive utilization [14, 31, 33]. Knowledge of family planning and access to media (TV or radio) strongly determined the use of contraceptives [34–36]. At the community level, factors like region, place of residence, community-level education, community-level religion, and community-level wealth are factors associated with modern contraceptive utilization [29, 33, 37, 38].

Investigating individual and community-level factors in family planning is critical to designing evidence-based programming to reach youth in greatest need [39]. It also helps policymakers and program managers identify the most vulnerable and marginalized populations [16]. Even though health statisticians and health planners neglected youth, this creates a gap in solving health issues specific to the youth population segment. Informing health planners about youth-specific concerns is crucial to addressing youth problems related to reproductive health [18, 40].

Ethiopia has identified family planning as a critical intervention in achieving the targets. Ethiopia made significant efforts to increase access to family planning information and a variety of family planning method options [2]. It is estimated that more than 90% of the Ethiopian population has access to modern family planning methods through community facilities, social marketing, and outreach based modalities. About 99% of facilities and 79% of health posts offer family planning services at least five days per week [41].

In Ethiopia, there is a high youth population that is affected by the practice of early marriage [42]. As a result, Ethiopia has implemented youth-friendly reproductive health education programs to increase reproductive health knowledge and prevent unsafe sex, unintended pregnancies, and abortions among youth [41]. This has contributed to improved contraceptive utilization. But still, contraceptive utilization is too low to achieve the national set of targets. As a result, the government must create an enabling environment for family planning among youth and increase funding for family planning in order for these people to meet set targets [2].

Contraceptive utilization has shown a remarkable increase over the last decade in Ethiopia, and its prevalence will increase from 4.3% to 44% in 2020 among the reproductive age group [43]. n Ethiopia, modern contraceptive prevalence among women aged 15–34 years was 57.4%, which was higher when compared to women aged 35–49 years [43].

Therefore, this study investigates the level modern contraceptive utilization and its associated factors among married women aged 15–24 and 25–34 years.

## Methods and materials

### Study setting and data source

A cross-sectional study design was conducted using secondary data analysis from the Ethiopia Mini Demographic Health Survey (EMDHS) 2019. EMDHS collect national and subnational representative data for urban and rural areas and for each of the nine regions and two administrative cities. EMDHS is the second cross sectional study conducted in Ethiopia. EMDHS is a nationally representative survey that evaluates contraceptives, maternal and child health, and a variety of health indicators and socio-demographics.

The data for this analysis were extracted from EMDHS 2019 and accessed from the Measure DHS website (http://www.dhsprogram.com). EMDHS 2019 data sets were downloaded in STATA format after getting permission from the Measure DHS website (http://www.dhsprogram.com). After downloading the required data, the data management and analysis have been carried out by STATA. The sample technique for EMDHS 2019 was selected using a stratified and two-stage cluster design. EMDHS 2019 used enumeration areas (EAs) as primary sampling units and households as secondary sampling units.

### Study design and participants

EMDHS 2019 uses 149,093 enumeration areas (EAs). The sample technique for EMDHS 2019 was selected using a stratified, two-stage cluster design. EMDHS 2019 used enumeration areas (EAs) as primary sampling units and households as secondary sampling units. In the first stage, a total of 305 EAs (93 in urban areas and 212 in rural areas) were selected with a probability proportional to EA size and with independent selection in each sampling stratum for the survey. Then, a fixed number of 30 households per cluster were selected with equal probability through systematic selection. Finally, the 2019 EMDHS survey covered 8,663 households out of the selected 8,794 households, providing a response rate of 99%.

**Fig 1:**
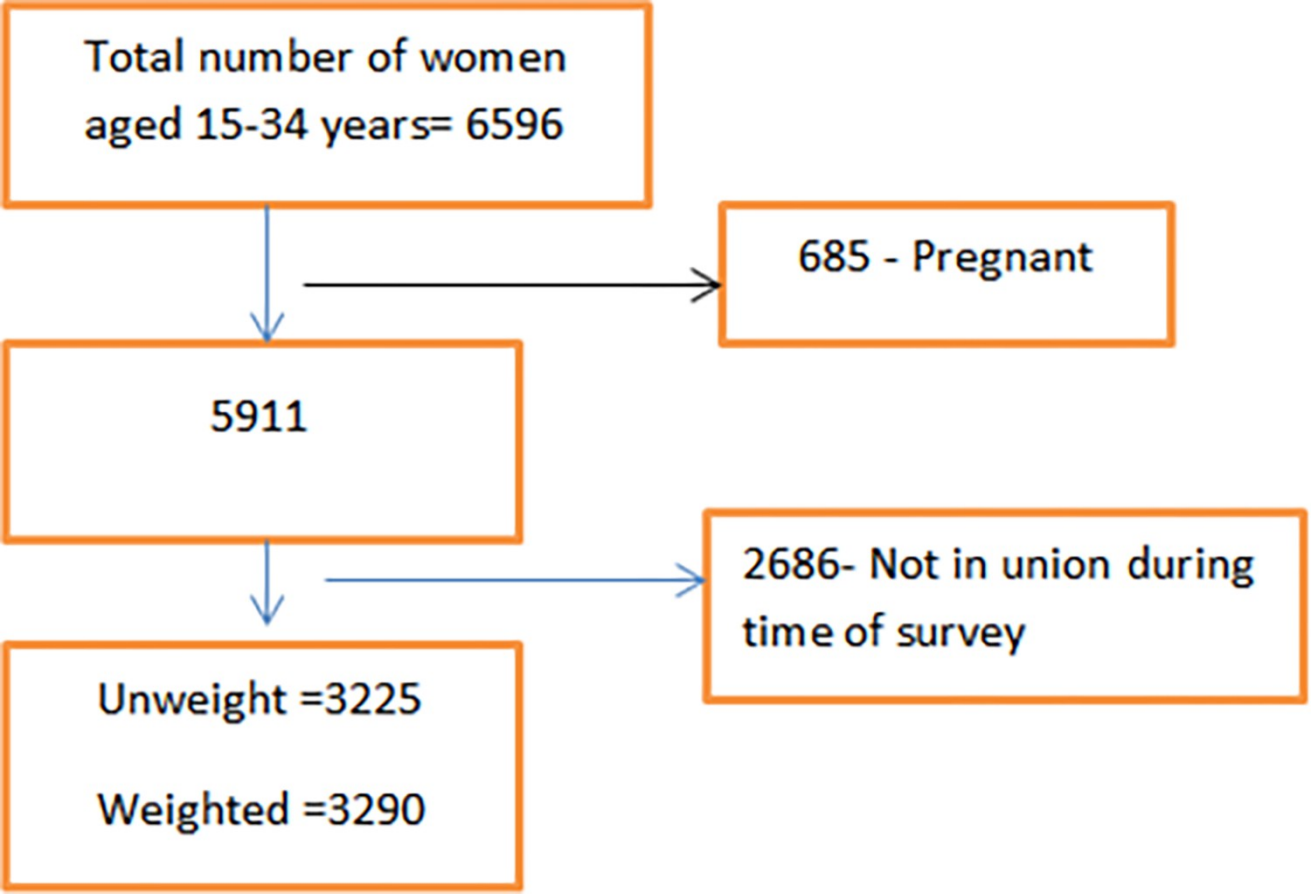
Schematic illustration of women aged 15–34 years included in the study. Pregnant women and women who were not current in union were excluded from the study. After weighting of sample, 3290 women aged 15–34 years were the final sample size of the study.

In the 2019 EMDHS survey, a total of women completed the interview out of 16,583 women identified for the interview. In this study, all married or unmarried non-pregnant women aged 15–34 years were included. The study excludes pregnant mothers and those who were not in union at the time of the survey. A total of 3290 weighted women aged 15–34 years were the final sample size of this study (Fig 1).

### Study variables

#### Dependent variable

The outcome of interest in this study was the current use of modern contraceptives among women aged 15–24 years and women aged 25–34 years. Modern contraceptive utilization is categorized as "1" for modern contraceptive users and ‘0’ for those not using modern contraceptives (women who have been utilizing traditional, folkloric or no method). Modern contraceptive users were those women who currently use any type of modern contraceptive method, such as injectable, implantable, oral contraceptive pills, female sterilization, male sterilization, intrauterine device (IUD), male and/or female condom, locational amenorrhea method, diaphragm, foam/ jelly, and other modern contraceptive methods (including cervical caps and contraceptive sponges) (41).

#### Independent variables

Individual and community level explanatory variables were used to assess modern contraceptives.

#### Individual level factors

Individual level factors include age at first birth (age less than 18 years, 18–24, 24+), educational level (no education, primary, secondary, higher), wealth index (poorest, poorer, middle, richer, richest), religion (Christianity, Muslim, protestants, others), parity (0, 1–3, 4+), number of living children (0, 1, 2, 3+), births in the last three years (no birth, one birth, two & above), family size (1–4, 5–7,8+), number of under five children in household (0, 1–2, 3+), access to TV or radio (no, yes), and knowledge on modern contraceptive method (no, yes).

#### Community-level variables

Community-level explanatory variables include community knowledge of modern contraceptives, community level of education, community religion afflation, community wealthy status, region (Tigray, Afar, Amhara, Oromia, Somali, Benishangul, SNNPR (Southern Nations, Nationalities, and Peoples), Gambela, Harari, Addis Ababa, and Dire Dawa) and place of residence (urban, rural).

EMDHS did not collect community-level / cluster-level data except place of residence and region. Therefore, these community-level data were generated by aggregating the individual characteristics of respondents. The aggregates were computed using the proportion of a given variable’s subcategory in a given cluster. The aggregate median value is used since the data is not normally distributed. Finally, the aggregated values were classified as low and high if the proportions of the clusters were below and above the national level, respectively.

Community-level religion: the proportion of Muslims was taken, with a cutoff value of 50%. Greater than 50% of Muslim women in the cluster were regarded as high community level Muslims, and less than 50% of Muslim women in the cluster were considered low community level Muslims.

Community-level wealth is the proportion of poor or poorest mothers within the cluster. Referencing to the national level, which was is 50%, classified into low if the value is above 48.9% and high if the value is below 50%.

Community women’s education is defined as the proportion of mothers who attended primary, secondary, or higher education within the cluster. Using the national level, which is 50%, the value is classified as low if it is below 50% and high if it is above 50%.

### Data processing and analysis

During DHS data analysis, sampling weight was applied to an individual interview unit of analysis to adjust for differences in probability of selection and interview between cases in a sample due to design, happenstance, or corrections for differential response rates. A sample weighing adjustment is needed to produce a proper representation of survey data. The data were checked for completeness, cleaned of missing values, and re-coded accordingly. Descriptive methods such as frequency, graph, and multilevel logistic regression analysis have been carried out by STATA version 16.

A two-level multilevel logistic regression model is appropriate to measure DHS data since the data is hierarchical in nature. It used to explore factors affecting modern contraceptive utilization at the individual and cluster level [44]. Four models were considered in the multilevel logistic regression; model one (Model I) was empty without an explanatory variable and presented the total variance in modern contraceptive utilization among clusters; model two (Model II) was adjusted for individual level variables; model three (Model III) was adjusted for cluster level variables; and model four (Model IV) was adjusted for individual and cluster level variables.

Variables with a *p*-value less than 0.2 at bivariable multilevel logistic regression analysis were candidates for a multivariable multilevel logistic regression model to identify independent variables of modern contraceptive utilization. *P* values less than 0.05 in multilevel logistic regression were considered statistically significant independent factors for modern contraceptive utilization. The association was measured by adjusted odds ratios with 95% confidence intervals.

The measurement of variation was identified using interclass correlation (ICC). The model for fitness diagnostics was checked using Deviance Information Criteria (DIC), Alkaile Information Criteria (AIC), and the log-likelihood test. A model with the highest log-likelihood test and lowest AIC was used to estimate the goodness of fit. Multicollinearity was checked by the variance inflation factor (VIF) at cut point of 10. As a result, variable "knowledge of modern contraceptives" had a VIF value of 12.5 so it was excluded from the model because of multicollinearity, and other variables’ VIF values were less than 3 [44].

### Ethical consideration

Registration on the DHS website (www.dhsprogram.com) and approval from the measure DHS were required to access the data. Prior to the actual interview, informed consent was obtained from the participants, their guardian, or household head. The data was only used for statistical reporting and analysis, as well as for the proposed research project. The data should be treated as confidential, and no effort should be made to identify any household or individual respondent interviewed in the survey.

## Result

### Socio-demographic characteristics

The EDHS 2019 interviewed 3290 married or currently in union women between the ages of 15 and 34 years. Among these, 1210 (36.7%) and 2080 (63%) women were in the age groups of 15–24 and 25–34 years, respectively. More than one third of women aged 15 to 24 and 25 to 34 were orthodox. The richest women were roughly one-third of those aged 15–24 and 25–34 years. Almost all women aged 15–24 and 25–34 had knowledge of modern contraceptive methods. More than half of women aged 15–24 and 25–34 had their first birth less than 18 years ago and had one birth in the last three years. Most women aged 15–24 and 25–34 had one or two children under five in their household. On the other hand, half of women aged 15–24 have one to three children. Almost one third of women aged 15–24 have no children.

Regarding community-level factors, more than half of women aged 15–24 and 25–34 lived in rural areas, high educated communities, and communities with high contraceptive knowledge (Table 1).

**Table 1:** Socio-demographic characteristics of women aged 15–24 and 25–34 years in EDHS 2019.

Variable	15–24 years		25–34 years	
	Weighted Frequency	Percentage	Weighted Frequency	Percentage
Individual level factor				
Age of respondent at first birth				
Less than 18 years	497	41.03	1147	55.16
18–24 years	342	28.27	765	36.81
25 and above			74	3.57
Women education level				
No education	249	20.56	1084	52.13
Primary	716	59.20	700	33.64
Secondary	179	14.83	181	8.71
Higher	66	5.42	115	5.52
Religion				
Orthodox	464	38.37	850	40.90
Muslin	394	32.57	630	30.29
Protestant	340	28.05	564	27.07
Other	12	1.00	36	1.74
Wealth index				
Poorest	228	18.88	368	17.69
Poorer	216	17.82	403	19.36
Middle	217	17.95	406	19.52
Richer	261	21.53	408	19.62
Richest	288	23.82	495	23.80
Birth in last three years				
No birth	524	43.29	781	37.55
One birth	607	50.17	1143	54.93
Two & above	79	6.54	156	7.52
Parity				
0	372	30.70	92	4.46
1–3	795	65.71	1073	51.54
4 and above	43	3.59	915	44.00
Number of living children				
No child	386	31.93	99	4.75
1 child	504	41.61	246	11.84
2 children	224	18.53	511	24.57
3 and above children	96	7.93	1224	58.83
Household size				
1–4	798	65.98	697	33.48
5–7	352	29.10	1248	60.01
8–14	60	4.92	135	6.52
Number of under five children in household				
None	347	28.66	397	19.09
1–2	810	66.98	1521	73.09
3 & above	53	4.36	162	7.81
Access to TV or Radio				
No	755	62.40	1288	61.92
Yes	455	37.60	792	38.08
Knowledge of modern contraceptive methods				
No	40	3.26	67	3.22
Yes	1170	96.74	2013	96.78
Community level factor				
Resident				
Urban	365	30.21	618	29.69
Rural	845	69.79	1462	70.31
Region				
Tigray	66	5.45	140	6.75
Afar	17	1.42	23	1.11
Amhara	276	22.83	489	23.51
Oromia	508	42.01	755	36.28
Somali	54	4.48	94	4.52
Benishangul-Gumuz	13	1.09	25	1.21
SNNPR	223	18.50	441	21.18
Gambela	7	0.59	10	0.46
Harari	3	0.23	6	0.29
Addis Ababa	33	2.72	87	4.20
Dire Dawa	9	0.68	10	0.50
Community level of education				
Low	133	11.03	868	41.75
High	1077	88.97	1212	58.25
Community level of wealth				
Low	402	33.27	646	31.05
High	807	66.73	1434	68.95
Community level of knowledge in community				
Low	19	1.54	10	0.46
High	1191	98.46	2070	99.54
Community level of religion				
Low	847	70	1462	70.31
High	336	30	618	29.69

### Prevalence and method of modern contraceptive utilization

**Fig 2:**
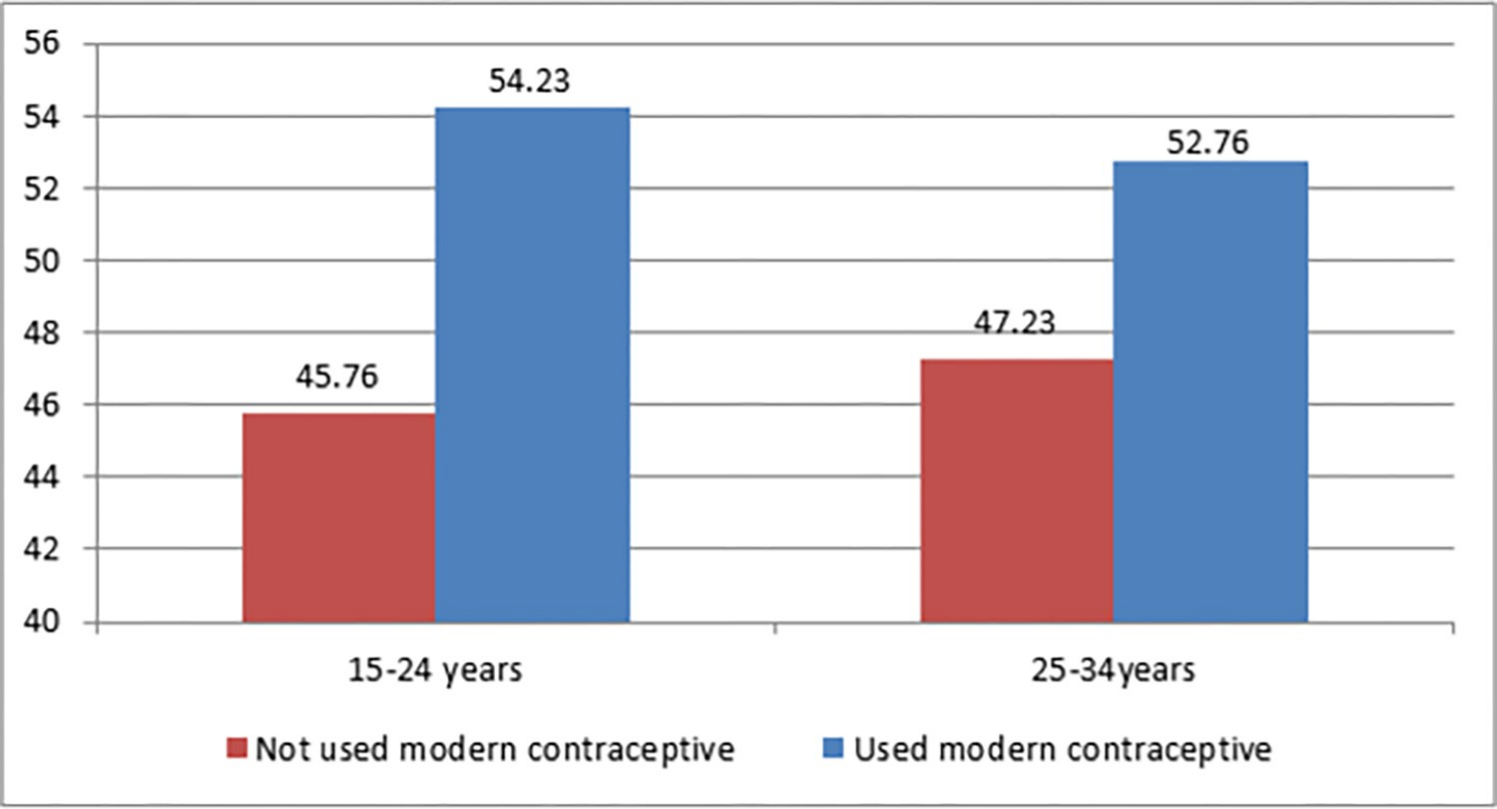
Proportion of modern contraceptive user among women aged 15–24 and 25–34 years in Ethiopia, EMDHS 2019.

**Fig 3:**
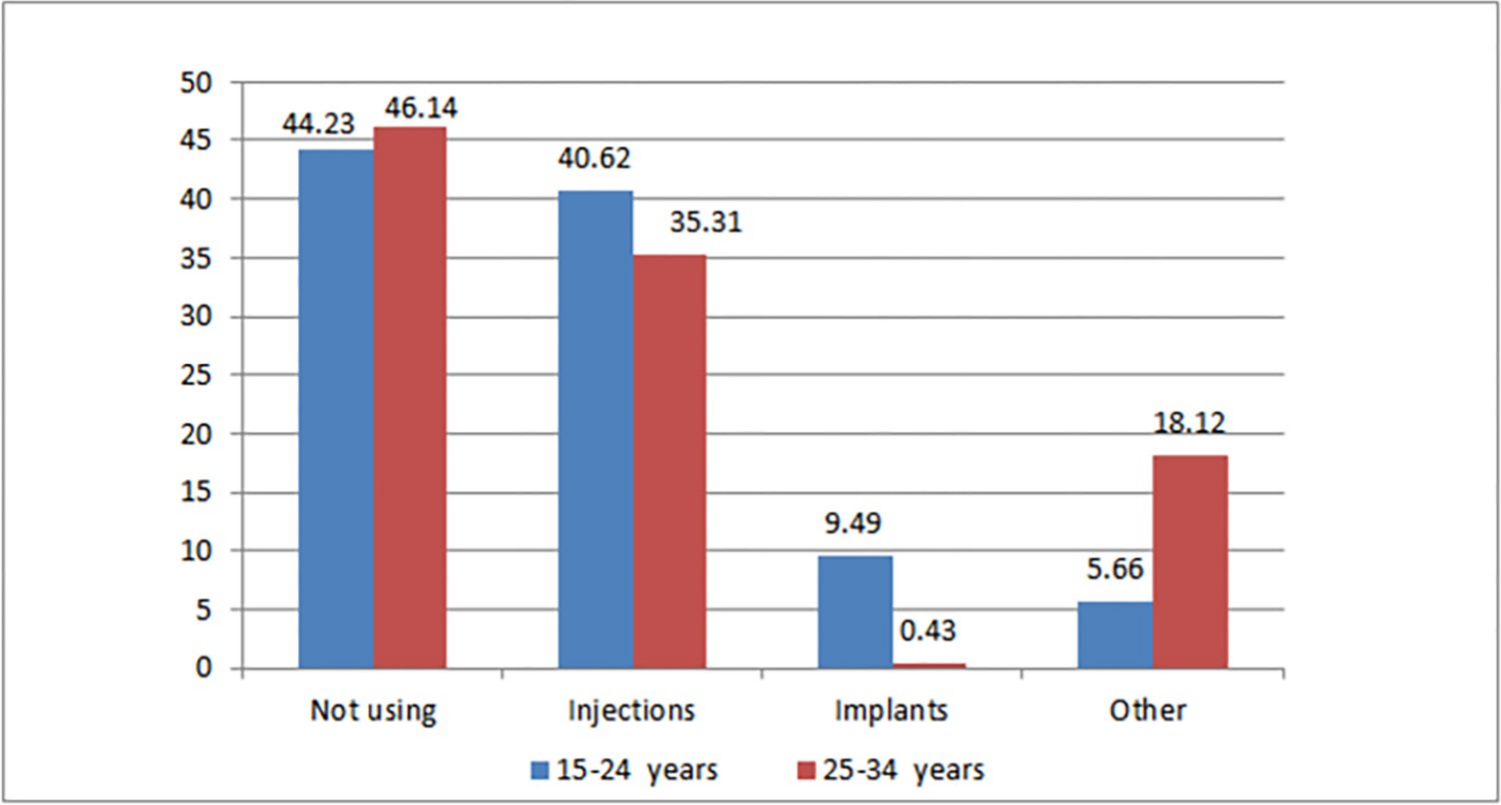
Proportion of modern contraceptive user by contraceptive method among women aged 15–24 and 25–34 years in Ethiopia, EMDHS 2019.

Modern contraception utilization among women aged 15 to 24 years was 54.23%. On the other hand, modern contraception utilization among women aged 25–34 years was 52.6% (Fig 2). Modern contraception utilization is slightly higher among women aged 15 to 24 years. Injection was the most commonly used contraceptive, used by 40.62% of women aged 15–24 years and 35.31% of women aged 25–34 years. The implant was the second most commonly used modern contraceptive (Fig 3).

### Determinant of modern contraceptive utilization

After adjusting for individual and community-level factors of modern contraceptives among women aged 15–24 years, variables with p <0.2, such as educational level of women, wealth index, religion, parity, births in the last three years, number of household members, number of living children in household, number of under five children in household, religion, access to radio or TV in household, region, place of residence, community level of wealth, community level of education, and community level of religion, were selected for multivariable multilevel analysis.

In multivariable multilevel analysis, the likelihood of using modern contraceptives among women aged 15–24 years was high among those who had primary education [AOR = 2.22; 95% CI: 1.0–3.83] and who had 3 and above children [AOR = 14.29; 95% CI: 1.61–126.25], when compared to women who had no formal education and no child, respectively. Women who are protestant [AOR = 0.29; 95% CI: 0.14–0.61] and who live in 5–7 households [AOR = 0.34; 95% CI: 0.17–0.69] were less likely to use modern contraceptives when compared to women who practiced orthodox religion and women who lived in up to four households, respectively. At the community level, women who live in Amhara [AOR = 6.98; 95% CI: 1.46–11.95], Benishangul-Gumuz [AOR = 4.54; 95% CI: 1.65–12.48], SNNPR [AOR = 6.42; 95% CI: 2.10–19.58], and Addis Abeba region [AOR = 4.76; 95% CI: 1.08–20.83] were more likely to use modern contraceptives when compared to Tigray region (Table 2).

**Table 2:** Multilevel logistic regression assessing effects of individual and community level characteristics on modern contraceptive utilization among women age 15–24 years in EDHS 2019.

Variable	Model 1 AOR [95%]	Model 2 AOR [95%]	Model 3 AOR [95%]	Model 4 AOR [95%]
Individual level factor				
Fixed effect				
Women education level				
No education		1		1
Primary		2.59**[1.32–5.09]		2.22*[1.02–4.83]
Secondary		3.25*[1.26–8.35]		2.77[0.96–7.95]
Higher		1.28[0.29–5.69]		1.12[0.25–5.59]
Religion				
Orthodox		1		1
Muslin		0.22*[0.10–0.45]		0.43[0.12–1.41]
Protestant		3.25**[1.26–8.35]		0.29**[0.14–0.61]
Other		1.28[0.29–5.69]		0.89[0.06–12.29]
Wealth index				
Poorest		1		1
Poorer		1.89[0.70–4,66]		1.56[0.53–4.56]
Middle		3.11*[1.04–9.28]		2.50[0.67–9.32]
Richer		1.97[0.68–5.68]		1.70[0.44–6.52]
Richest		2.35[0.73–7.57]		2.19[0.43–11.06]
Birth in last three years				
No birth		1		1
One birth		0.89[0.41–1.92]		0.94[0.43–2.05]
Two & above		0.53[014–1.96]		0.65[0.16–2.59]
Parity				
0		1		1
1–3		3.11[0.42–22.73]		2.97[0.44–19.91]
4 and above		0.84[0.06–10.38]		0.82[0.06–10.18]
Number of living children				
No child		1		1
1 child		2.47[0.35–17.19]		2.49[0.38–16.34]
2 children		3.61[0.53–24.18]		3.84[0.59–25.03]
3 and above children		13.17*[13.17–119.21]		14.29*[1.61–126.25]
Household size				
1–4		1		1
5–7		0.34[0.17–0.68]		0.34**[0.17–0.69]
8–14		1.69[0.54–5.26]		2.04[0.62–6.72]
Number of under five children in household				
None		1		1
1–2		1.09[0.40–2.93]		1.10[0.40–3.01]
3 & above		0.26[0.05–1.21]		0.27[0.05–1.35]
Access to TV or radio				
No		1		1
Yes		1.48[0.68–3.21]		1.51[0.67–3.39]
Community level factor				
Resident				
Urban			1	1
Rural			0.89[0.51–1.53]	1.01[0.49–2.41]
Region				
Tigray			1	1
Afar			0.64[0.26–1.55]	1.32[0.36–4.99]
Amhara			2.86*[1.47–5.54]	6.98**[2.30–21.16]
Oromia			1.79[0.95–3.36]	4.17*[1.46–11.95]
Somali			0.08*[0.01–0.54]	0.17[0.01–1.86]
Benishangul-Gumuz			2.33*[1.19–4.54]	4.54**[1.65–12.48]
SNNPR			2.17*[1.10–4.30]	6.42**[2.10–19.58]
Gambela			0.88[0.32–2.41]	1.83[0.40–8.22]
Harari			1.48[0.62–3.56]	2.65[0.70–10.02]
Addis Ababa			2.23[0.78–6.38]	4.76*[1.08–20.83]
Dire Dawa			1.13[0.55–2.35]	2.50[0.80–7.82]
Community level of education				
Low			1	1
High			1.12[0.70–1.78]	1.24[0.48–3.20]
Community level of wealth				
Low			1	1
High			1.33[0.88–2.00]	1.05[0.50–2.18]
Community level of religion				
Low			1	1
High			0.4***[0.27–0.67]	0.47[0.14–1.56]
Random effect				
ICC	31%	17%	13%	11%
Variance	1.05	0.97	0.51	0.49
Model fitness				
Log likelihood	-788	-742	-734	-728
AIC	1581	1517	1358	1340
BIC	1592	1590	1485	1450

AOR = adjusted Odds Ratios; CI = Confidence Interval; 1 = Reference Category.

*p*<* 0.05

**p*<* 0.01

***p*<* 0.001

Regarding women aged 25–34 years, individual and community level variables such as educational level of women, wealth index, religion, parity, number of living children in household, number of under five children in household, access to radio or TV in household, religion, region, and place of residence, community level of wealth, community level of education, and community level of religion were selected for multivariable multilevel analysis.

In multivariable multilevel analysis, the likelihood of using modern contraceptives among women of reproductive age was high among those who had one or two under five children in the household [AOR = 1.66; 95% CI: 1.03–2.68] when compared to women who had no under five children in the household. At the community level, women who lived in Amhara [AOR = 3.54; 95% CI: 1.79–6.97], Oromo [AOR = 2.10; 95% CI: 1.09–4.03], Benishangul-Gumuz [AOR = 4.58; CI: 1.04–4.39], and SNNPR [AOR = 2.31; 95% CI: 1.10–4.83] are more likely to use modern contraceptives when compared to women who lived in Tigray region (Table 3).

**Table 3:** Multilevel logistic regression assessing effects of individual and community level characteristics on modern contraceptive utilization among women age 25–34 years in EMDHS 2019.

Variable	Model 1 AOR [95%]	Model 2 AOR [95%]	Model 3 AOR [95%]	Model 4 AOR [95%]
Individual level factor				
Fixed effect				
Women education level				
No education		1		1
Primary		1.55[1.00–2.42]		1.42[0.91–2.23]
Secondary		1.41[0.77–2.58]		1.42[0.77–2.64]
Higher		2,30[0.86–6.10]		2.36[0.85–6.53]
Religion				
Orthodox		1		1
Muslin		0.31***[0.19–0.50]		0.58[0.24–1.38]
Protestant		0.74[0.47–1.18]		0.72[0.42–1.24]
Other		0.64[0.22–1.83]		0.60[0.20–1.79]
Wealth index				
Poorest		1		1
Poorer		1.10[0.64–1.88]		0.83[0.47–1.46]
Middle		1.70[0.89–3.26]		1.19[0.60–2.39]
Richer		1.26[0.60–2.65]		0.83[0.37–1.84]
Richest		1.38[0.64–2.97]		0.74[0.30–1.82]
Parity				
0		1		1
1–3		5.51[0.55–54.56]		6.35[0.66–60.69]
4 and above		5.38[0.52–55.20]		6.21[0.62–61.46]
Number of living children				
No child		1		1
1 child		.0.76[0.08–7.16]		0.62[0.07–5.53]
2 children		1.10[0.13–9.08]		0.88[0.11–6.85]
3 and above children		0.92[0.11–7.49]		0.76[0.10–5.83]
Number of under five children in household				
None		1		1
1–2		1.54[0.95–2.50]		1.66*[1.03–2.68]
3 & above		1.65[0.43–6.22]		0.63[0.29–1.39]
Community level factor				
Resident				
Urban			1	1
Rural			0.66[0.44–1.00]	0.63[0.39–1.02]
Region				
Tigray			1	1
Afar			0.35*[0.14–0.85]	0.52[0.20–1.34]
Amhara			2.71***[1.47–4.98]	3.54***[1.79–6.97]
Oromia			1.43[0.86–2.38]	2.10*[1.09–4.03]
Somali			0.14*[0.04–0.41]	0.24[0.07–1.80]
Benishangul-Gumuz			1.68[0.92–3.09]	2.14*[1.04–4.39]
SNNPR			1.53[0.86–2.71]	2.31*[1.10–4.83]
Gambela			0.58[0.25–1.37]	0.81[0.29–2.28]
Harari			1.13[0.57–2.25]	1.42[0.66–3.02]
Addis Ababa			1.08[0.60–1.93]	1.53[0.76–3.05]
Dire Dawa			0.88[0.43–1.83]	1.22[0.54–2.74]
Community level of religion				
Low			1	1
High			0.45*[0.28–0.72]	0.64[0.27–1.49]
Community level of education				
Low			1	1
High			1.63*[1.05–2.53]	1.43[0.90–2.28]
Community level of wealth				
Low			1	1
High			1.40[0.88–2.22]	1.31[0.76–2.26]
Random effect				
ICC	36%	12%	10%	9%
Variance	1.3	0. 82	0.62	0.60
Model fitness				
Log likelihood	-1309	-1223	-1248	-1197
AIC	2623	2489	2429	2414
BIC	2635	2627	2619	2610

AOR = adjusted Odds Ratios; CI = Confidence Interval; 1 = Reference Category.

*p*<* 0.05

**p*<* 0.01

***p*<* 0.001

### Measure of variation

The random effects of multilevel logistic regression analysis indicated that there was a significant variation in using modern contraceptives across the clusters. The intra-cluster correlation coefficient (ICC) was 31% and 36% among women aged 15–24 years and 25–34 years, respectively, which attributed to the variation between intra-clusters. It decreases successively in model in both age groups. Hence, the combined models of individual-level and community-level factors were preferred for predicting contraceptive use. The model of fitness, which has the highest log likelihood and the lowest deviance, indicated that model IV is the best-fitted model (Tables 2 and 3).

## Discussion

Improving family planning has a significant impact on health by lowering child and maternal mortality. To increase family planning utilization, it is crucial to consider age variation. This study was based on the data from a nationally representative survey of women aged 15–34 years.

The prevalence of modern contraceptive utilization among women aged 15–24 and 25–34 years was 54.23% and 52.6%, respectively. This study also showed slightly higher modern contraceptive use among women aged 15–24 than among women aged 25–34. The finding reported the use of modern contraceptive use among women aged 15–24 was higher than women aged 25–34. This study was consistence with studies done in Bangladesh [45], Indonesia [46] and Ethiopia [47]. This could be explained by young women’s desire to avoid pregnancy because they might want to continue their education and they may be too young to look after the baby [48].

Finding from various studies indicated that prevalence of contraceptive decrease as the age of women increases. The possible reason might be.But this finding was contradict with studies done in Malawi [49], Ghana [50], and Multicounty [51].

The prevalence of modern contraceptive utilization among women aged 15–24 and 25–34 years was 54.23% and 52.6%, respectively. This study showed slightly higher modern contraceptive use among women aged 15–24 than among women aged 25–34. This study was supported by studies done in Indonesia (43) and Ethiopia [47] and Multicounty study [51]. This might be due to the fact that they are more mature, have better knowledge of family planning, have obtained a better education, and have an occupation [52].

This study finding shows the significant difference of factor associated with modern contraceptive utilization between women age group 15–24 years and 25–34 years. Significant specific factors associated with modern contraceptive use among women aged 15–24 years were primary education, women who have three or more children, Protestants, and women who live in five to seven households. Whereas a specific factor associated with modern contraceptive use among women aged 25–34 years was the number of under five children in the household. But in this studies region was common significant variable for both age groups. This finding highlight community level factor like region may influence the community as whole.

Geographical region was the only predictor of modern contraceptive utilization for both women aged 15–24 and 25–34 years. Regions (Amhara, Oromia, Benishangul-Gumuz, and SNNPR) were a factor affected modern contraceptive utilization for both age groups. Addis Abeba influence modern contraceptive utilization among women aged 15–24 but not significant for women aged 25–34 years. Generally, Amhara, Oromia, Benishangul-Gumuz, and SNNPR regions are more likely to use modern contraceptives than Tigray regions. It is supported by studies done in Uganda [14], Nigeria [31], Ethiopia [53] and Cameron [54]. This could be due to the fact that some regions have higher fertility than others [55]. Moreover, this implies that differences in the socio-demographic characteristics of respondents, variations in the availability and accessibility of different family planning services, and cultural, religious, and community awareness about family planning are possible explanations for regional variations of modern contraceptives [56–58].

Primary educated women aged 15–24 are more likely to use modern contraceptives when compared to uneducated women aged 15–24. This study is consistent with findings from Ghana [50], multicounty [51] and Gondar [59], which indicated highly educated women are more likely to use modern contraception. This is due to educated women’s increased exposure to reproductive health information and better access to family planning services by removing different myths, misconception and misuse of contraceptives use [60]. Education empowers women to decide family planning related issues independently which enable women to make informed choices about appropriate method. Through education women know where to find available contraceptive methods and to understand the positive impacts of contraceptives [50, 61]. Educated women want to limit their number of children because of employment [46].

Regarding religious women, protestant women were less likely to use modern contraceptives when compared to orthodox women aged 15–24 years. On the contrary, studies done in Ethiopia, Ghana, and Tanzania [28, 62, 63] indicated that Protestants were more likely to use modern contraceptives. This could be due to the fact that religion has a significant influence on a wide range of social attitudes, but the relationship between religion and its perspective on contraception has largely gone unexplored [64, 65]. This might be due to the belief that family planning is incompatible with religious beliefs. Personal interpretations of family planning positions in religion also affect the use of modern contraceptives [63].

Women who had more than three living children in their household were more likely to use modern contraception when compared to women who had no children in their household. The more births a woman has, the more likely she is to use modern contraception. This finding is supported by studies in Senegal [38] and Ethiopia [47]. This implies that the likelihood of wanting no more children increases with the actual number of living children. The possible reasons might be that women who had more children might have a greater need to space or limit births than those who had no children. Additional reasons might be financial issues, a desire to upgrade their education, a desire for career development, or participation in the labour market [64, 65].

Family size is negatively associated with modern contraceptive utilization in women aged 15–24 years. This study was agreed with studies done in Ethiopia [66] and India [67]. This might be due to mothers with large families having less intention to use family planning because of a negative attitude towards contraception [68]. Studies also suggest women might face pressure from their partners or the community to have more children later [69]. Other reasons might also be religious beliefs, cultural practices, misconceptions, health concerns, and infrequent sex [68, 70, 71]. However, previously study showed that this finding has disagreement with studies conducted in Ethiopia [72] and Indonesia [46].

Women who had one to two under five children in their household were more likely to use modern contraceptive utilization in women aged 25–34 years when compared to women who had no under five children in their household. Study done in another part of Ethiopia supported this evidence [72] and Liberia [73]. The reason might be that women who have a desired number of children are more likely to use contraceptives. If there are under five children in the household, women tend to use contraceptives for spacing until the children grow [68, 74].

## Conclusion

In conclusion, more than 50% of respondents use modern contraceptives, but it is lower than the national level target (67%). Individual and community level factors influence the use of modern contraceptives in both age groups. Region is a common and significant variable for both age groups. However, specific factors associated with modern contraceptive use among women aged 15–24 years are primary education, women who have three or more children, Protestants, and women who live in five to seven households. A specific factor associated with modern contraceptive use among women aged 25–34 years was the number of under five children in the household.

Modern contraceptive use is a valuable indicator for national family planning programmes because it shows how well they are achieving a key mission. Therefore, interventions to improve family planning should consider the level of modern contraceptive use by age group, region, educational status, religion, and fertility level in the planning of family planning programmes. Policymakers need to understand specific factors that affect modern contraceptive use.

### Study and limitation of study

The EMDHS 2019 survey was a population-based study that achieved a large sample size. This improves the study’s power and enables the generalizability of the findings of modern contraceptive use among women aged 15–34 years. However, the EMDHS 2019 survey was a cross-sectional study, so this study cannot analyses the cause-and-effect relationship between factors and modern contraceptive use. The data collected is based on women’s self-reporting and might be subject to recall bias. The EMDHS 2019 survey does not include some variables related to contraception. It also lacks data on cultural issues, infrastructure, service quality, and policies in Ethiopia.

## Supporting information

S1 File (DTA)

## List of abbreviations

FP: family planning
SNNP: Southern Nations, Nationalities and Peoples
DHS: Demographic and Health Survey
EMDHS: Ethiopian Mini Demographic and Health Survey
